# Women’s Perceptions of Using Mobile Phones for Maternal and Child Health Support in Afghanistan: Cross-Sectional Survey

**DOI:** 10.2196/mhealth.9504

**Published:** 2018-04-10

**Authors:** Fazal Yamin, Jaranit Kaewkungwal, Pratap Singhasivanon, Saranath Lawpoolsri

**Affiliations:** ^1^ Department of Tropical Hygiene Faculty of Tropical Medicine Mahidol University Bangkok Thailand

**Keywords:** Afghanistan, mobile health, maternal health, child health, perception, mobile phone

## Abstract

**Background:**

Growing rates of global mobile subscriptions pave the way for implementation of mobile health (mHealth) initiatives, especially among hard-to-reach populations.

**Objective:**

This study aimed to determine the perceptions of Afghan women regarding the use of mobile phones for maternal and child health services.

**Methods:**

A cross-sectional survey was conducted in both rural and urban districts of Nangarhar Province, Afghanistan. The interviewer-administered questionnaire was used to assess participants’ demographic profile, mobile phone usage, and perception of respondents toward different aspects of health care delivery via mobile phones.

**Results:**

Of the 240 participants, 142 (59.2%) owned mobile phones and 220 (91.7%) routinely used mobile phones. Approximately 209 (87.1%) of participants were willing to receive health messages via a mobile phone. Automated voice call was the most preferred method for sending health messages. More than 90% of the women reported that they would like to receive reminders for their children’s vaccinations and antenatal care visits.

**Conclusions:**

Users’ perception was associated with mobile phone ownership, literacy level, and experience using mobile phones. In the study area, where the literacy rate is low, mHealth was well perceived.

## Introduction

Mobile technology has been treated as a necessity in both the developed and developing world. The high demand for this technology has resulted in exponential growth in the number of cellular subscribers worldwide. In 2014, there were more than 7 billion subscribers, with an average penetration rate of 96% [1].

Given the unprecedented growth in the use of mobile technology, much research and increasing resources have been devoted to the development of its application in various domains beyond its core functionality of telecommunication. The use of mobile phone devices to support medical and public health practices has led to the emergence of a new field of eHealth known as mobile health (mHealth) [2]. mHealth can be used for complex applications for health; however, simple health support applications using mobile phones’ core functionality have also been developed [2].In Afghanistan, a postconflict country, there were no mobile network operators until 2003; now there are 5 mobile network operators providing services to more than 20 million subscribers with a penetration rate of 83% covering 90% of the population [3]. A lack of landline telephones in most areas, high demand, a competitive market, and decreasing costs of mobile devices and services have all contributed to the rapid growth of mobile telephone services in the country.

Although gender inequality in Afghanistan has been improving in the past decade, accessibility to education and health care remains a great challenge among Afghan women. The national literacy rate for adult females was only 17% in 2012 [4]. Despite the limited access to education and health, a survey among Afghan women has shown that about 80% of women have access to a mobile phone [5]. This high rate of mobile phone use among Afghan women suggests that application of mHealth could be an effective method to promote maternal and child health.

Maternal and child health is a key priority area for the Ministry of Public Health of Afghanistan. The maternal mortality rate has been decreased from 1600 per 100,000 live births in 1990s to 327 per 100,000 live births in 2010 [6,7]. However, maternal mortality and child mortality rates in Afghanistan are still high compared with those in other countries. Lack of information is an important factor that contributes to poor health outcomes [8]. People in rural areas may not have access to many sources of information; for years, radio had been the only reliable medium for transferring information to the rural population.

Studies have shown that mobile phones can be used as an effective means for disseminating health-related messages to target populations. A study in India showed that mobile phones can be considered as an acceptable means of health care delivery [9]. Studies also show positive perceptions toward using mobile phone for health purposes; however, some important factors need to be considered before designing and implementing such a program, including user preference of receiving messages, tone and frequency of messages, and privacy and confidentiality [2,9-13].

This study aimed to determine the perceptions of Afghan women regarding the use of mobile phones for supporting maternal and child health, and the associated factors with those perceptions. This preintervention study could provide viable information in terms of contextualization for the development of appropriate mHealth initiatives.

## Methods

### Study Design

This cross-sectional survey was conducted in two study sites (rural and urban) in Nangarhar Province, Afghanistan, from August 2015 to September 2015. Nangarhar Province, consisting of 22 districts, is one of the largest provinces in the country. Women of child-bearing age (18-49 years) who resided in the study areas and who agreed to participate in our study were considered eligible for the survey.

A sample size of 240 (120 per study site) was calculated with expected women’s perceptions toward utilization of mHealth of 80% with 5% error and 80% power. A multi-stage stratified random cluster sampling was performed to select the study sites. First, the 22 districts were classified as rural or urban areas. One district in an urban area (Jalalabad City) and 2 districts in a rural area (Rodat and Kama) were randomly selected. Then, 1 subdistrict per district in the rural area and 2 subdistricts in the city were randomly chosen. Finally, 2 villages or sectors in each subdistrict (total of 8 villages/sectors) were randomly selected for data collection.

Systematic sampling was used to select households in each village. Finally, random sampling was used for selecting 1 interviewee in each household.

The interviewer-administered questionnaire used in this study was adopted from a similar study conducted in India [9]. The questionnaire was reviewed by 3 experts before implementation. The interviewers were trained for data collection. The questionnaire assessed participants’ demographic profile, mobile phone usage, and perception of respondents toward different aspects of health care delivery via mobile phones.

### Data Analysis

Data were analyzed using Statistical Package for the Social Sciences (SPSS) version 18 (SPSS Inc, Chicago, IL). Frequencies, means, and SD were used to describe the variables. Demographic and mobile phone usage data were compared between respondents in rural and urban areas. Chi-square and binary logistic regression models were used to identify factors associated with perceptions and willingness to use mobile phones for health. Multiple logistic regression was performed to determine adjusted odds ratio (OR) for the perceptions and willingness to use mobile phones for health; the adjusted variables included residence, ability to read, ownership of mobile phone, routine use of mobile, age group, and duration of mobile phone use.

### Ethical Statement

The study was approved by the Ethics Committee of the Faculty of Tropical Medicine, Mahidol University, Thailand, and the Institutional Review Board of the Ministry of Public Health, Afghanistan. Information about the study was provided to participants, and informed consent was obtained from each participant.

## Results

### Characteristics of Participants

A total of 240 women participated in the study (120 participants from urban areas and 120 from rural areas). The mean household size was 7.1 individuals with an SD of 3.0 (minimum 2, maximum 14). Most households (228/240, 95%) had at least 1 mobile phone. Few participants were employed, and 89.6% (215/240) were housewives. Only one fourth of the women (57/240) were able to read, and among those, 33 had attended community-based schools (Table 1). These characteristics were not significantly different between women in the rural area and those in the urban area. Approximately, 47.5% (57/120) and 70.8% (85/120) of women in rural and urban areas, respectively, owned a mobile phone. However, most of the women (220/240, 91.7%) routinely used mobile phones for making calls. The main reason for not using mobile phones was the cost.

**Table 1:** Demographic characteristics of the study population (N=240).

Characteristics		n (%)
**Living area**		
	Rural	120 (50.0)
	Urban	120 (50.0)
**Ability to read**		
	No	183 (76.2)
	Yes	57 (23.8)
**Education level**		
	No education	183 (76.2)
	Informal education (community-based study)	33 (13.8)
	Primary and secondary	9 (3.8)
	High school	15 (6.2)
**Occupation**		
	Housewife	215 (89.6)
	Teacher	11 (4.6)
	Officer	4 (1.7)
	Other	10 (4.1)
**Have children**		
	No	13 (5.4)
	Yes	227 (94.6)

Women in both rural and urban areas had, on average, approximately 4 years of experience in using mobile phones. Most of the respondents (154/220, 70.0%) were familiar with both the voice call and short message service (SMS) function; however, only 24.1% (53/220) of them used the SMS function. Overall, only 16.8% (37/220) of women were familiar with voice calls, SMS, and interactive voice response (IVR) functions. Mobile phones were commonly used for alarm setting (for prayers or waking up) and listening to the radio. Only 2 respondents from urban areas used the mobile phone alarm feature as a reminder for taking medication (Table 2).

### Perceptions Toward Using Mobile Phones for Health

About 72.1% (173/240) of our respondents thought that mobile phones could be used for health support. There was no significant difference regarding the perception of using a mobile phone for health purposes between women in rural and urban areas. Of these 173 respondents, 80.3% (n=139) and 75.7% (n=131) thought that mobile phones could be used for making appointments and counseling purposes, respectively, whereas 19.1% (n=33) reported that it could be used as a means for disseminating health-related information, and 8.7% (n=15) suggested that mobile phones could be used for treatment or vaccination reminders. According to responses to open-ended questions, other mobile phone purposes were to communicate with pharmacists regarding medication, receiving updates about hospitalized relatives, and receiving lab results from the hospital (Table 3).

Perceptions toward using mobile phones to support health were significantly associated with literacy, ownership and usage of a mobile phone, and age. Women who were able to read were 20 times more likely to agree that mobile phones can be used to support health compared with illiterate women. Those who owned or routinely used a mobile phone were about twice more likely to use a mobile phone for health support; however, this association was not significant after adjusting for other variables. In addition, younger women (aged ≤31 years) were 3 times more likely to perceive the usefulness of using a mobile phone for health purposes. However, this perception was not significantly different among women in rural areas and those in urban areas (Table 4).

**Table 2:** Characteristics of mobile phone use (N=240). IVR: interactive voice response. SMS: short message service.

Characteristics		Study site		Total	*P* value
		Rural (n=120)	Urban (n=120)		
**Ownership of mobile phone, n (%)**					
	No	63 (52.5)	35 (29.2)	98 (40.8)	
	Yes	57 (47.5)	85 (70.8)	142 (59.2)	<.001
**Routine use of mobile phone, n (%)**					
	No	14 (11.7)	6 (5.0)	20 (8.3)	
	Yes	106 (88.3)	114 (95.0)	220 (91.7)	.06
Duration of mobile use (years), mean (SD)		4.27 (1.99)	4.41 (1.91)	4.35 (1.94)	.49
**Familiarity with mobile functions, n (%)**					
	Voice call	14 (13.2)	15 (13.2)	29 (13.2)	.99
	Voice call and SMS	74 (69.8)	80 (70.2)	154 (70.0)	
	Voice call, SMS, and IVR	18 (17.0)	19 (16.6)	37 (16.8)	
**Using mobile phone for SMS, n (%)**					
	No	82 (77.4)	85 (74.6)	167 (75.9)	
	Yes	24 (22.6)	29 (25.4)	53 (24.1)	.62
**Using mobile phone as alarm, n (%)**					
	No	77 (72.6)	70 (61.4)	147 (66.8)	
	Yes	29 (27.4)	44 (38.6)	73 (33.2)	.07
**Using mobile phone alarm (n=73), n (%)**		**n=29**	**n=44**		
	For prayers	15 (51.7)	20 (45.5)	35 (47.9)	
	For waking up	12 (41.4)	20 (45.5)	32 (43.7)	
	For taking medicine	0 (0)	2 (4.5)	2 (2.7)	
	Other	12 (41.4)	17 (38.5)	29 (39.7)	
**Using mobile phone for other functions (n=167), n (%)**		**n=81**	**n=86**		
	Radio	65 (80.2)	71 (82.6)	136 (81.4)	
	Audio (MP3 and/or MP4)	18 (22.2)	16 (18.6)	34 (20.4)	
	Games	19 (23.5)	21 (24.4)	40 (24.0)	
	Camera	14 (17.3)	18 (20.9)	32 (19.2)	
	Internet	0 (0)	9 (10.5)	9 (5.4)	
	Calculator	10 (12.3)	12 (14.0)	22 (13.2)	
	Torch or flash light	53 (65.4)	47 (54.7)	100 (59.9)	
**Reasons for not using mobile phone (n=20), n (%)**		**n=14**	**n=6**		
	Lack of money	12 (86)	4 (67)	16 (80)	
	Lack of permission	7 (50)	2 (33)	9 (45)	
	Unable to use mobile phone	4 (29)	2 (33)	6 (30)	

**Table 3:** Perceptions toward using mobile phones for health purposes. SMS: short message service.

Perceptions		Study site		Total, n (%)	*P* value
		Rural (n=120), n (%)	Urban (n=120), n (%)		
**Do you think mobile phones can be used for supporting health?**					
	No	39 (32.5)	28 (23.3)	67 (27.9)	
	Yes	81 (67.5)	92 (77.7)	173 (72.1)	.11
**Ways mobile phones can be used for supporting health**					
	As a mean for disseminating health-related information	9 (11.1)	24 (26.1)	33 (19.1)	
	As treatment or vaccination reminder	8 (9.9)	7 (7.6)	15 (8.7)	
	For making appointments	64 (79.0)	75 (81.5)	139 (80.3)	
	For counseling	62 (76.5)	69 (75.0)	131 (75.7)	
	Other	6 (7.4)	6 (6.5)	12 (6.9)	
**Openness to receiving health advice via a mobile phone**					
	No	20 (16.7)	11 (9.2)	31 (12.9)	
	Yes	100 (83.3)	109 (90.8)	209 (87.1)	.08
**Topics of interest**					
	Nutrition	30 (25.0)	36 (30.0)	66 (27.5)	
	Breast feeding	75 (62.5)	71 (59.2)	146 (60.8)	
	Pregnancy	95 (79.2)	95 (79.2)	190 (79.2)	
	Vaccination	105 (87.5)	82 (68.3)	187 (77.9)	
	Hygiene	35 (29.2)	40 (33.3)	75 (31.3)	
	Other	16 (13.3)	24 (20.0)	40 (16.7)	
**Preferred frequency for receiving health information**					
	Daily	39 (32.5)	22 (18.3)	61 (25.4)	.03
	Weekly	57 (47.5)	75 (62.5)	132 (55.0)	
	Monthly	24 (20.0)	23 (19.2)	47 (19.6)	
**Preferred method for receiving health information**					
	SMS	3 (2.5)	12 (10.0)	15 (6.3)	.009
	Automated voice call	96 (80.0)	98 (81.7)	194 (80.8)	
	No preference	21 (17.5)	10 (8.3)	31 (12.9)	
**Willingness to use a free health call center/** **help** **-** **line number**					
	No	28 (23.3)	19 (15.8)	47 (19.6)	
	Yes	92 (76.7)	101 (84.2)	193 (80.4)	.14
**Openness to receiving reminders about National Immunization Days**					
	No	35 (29.2)	32 (26.7)	67 (27.9)	
	Yes	85 (70.8)	88 (73.3)	173 (72.1)	.67
**Openness to receiving reminders for children’** **s vaccinations**					
	No	9 (7.5)	16 (13.3)	25 (10.4)	
	Yes	111 (92.5)	104 (86.7)	215 (89.6)	.14
**Openness to receiving reminders for tetanus vaccination**					
	No	7 (5.8)	9 (7.5)	16 (6.7)	
	Yes	113 (94.2)	111 (92.5)	224 (93.3)	.61


**Openness to receiving reminders for antenatal/** **perinatal care visits**					
	No	9 (7.5)	6 (5.0)	15 (6.3)	
	Yes	111 (92.5)	114 (95.0)	225 (93.8)	.42
**Preferred time for receiving reminders**					
	On the day	5 (4.4)	5 (4.5)	10 (4.4)	.34
	1 day before	37 (32.5)	49 (44.1)	86 (38.2)	
	2 days before	67 (58.8)	53 (47.7)	120 (53.3)	
	1 week before	5 (4.4)	4 (3.6)	9 (4.0)	
**Preferred method for receiving reminders (n=235)**		**n=120**	**n=115**		
	Voice call	96 (80.0)	92 (80.0)	188 (80.0)	.01
	SMS	3 (2.5)	12 (10.4)	15 (6.4)	
	No preference	21 (17.5)	11 (9.6)	32 (13.6)	

### Mobile Phone Usage for Health Promotion

The majority (209/240, 87.1%) of our respondents were open to receiving health advice via mobile phones. Only 31 women (31/240, 12.9%) refused to receive health information. Those who refused were asked for the reason in an open-ended question. Among those women, 10 cited cultural restrictions, and one stated, “I don’t need it as we have health professionals in our family.”

The percentages of respondents’ preferred topics were as follows: pregnancy, 79.2% (190/240); vaccination, 77.9% (187/240); breast feeding, 60.8% (146/240); hygiene, 31.3% (75/240); and nutrition, 27.5% (66/240). Other suggested topics included family planning, infertility causes, and information on specific diseases, such as diarrhea, hepatitis B, malaria, and human immunodeficiency virus/acquired immune deficiency syndrome (HIV/AIDS). More than half of the respondents (132/240, 55.0%) preferred to receive the information on a weekly basis, whereas 25.4% (61/240) preferred to receive it on a daily basis, and 19.6% (47/240) preferred monthly. In terms of the method for receiving health information, the majority (194/240, 80.8%) of respondents preferred automated voice calls, whereas only 6.3% (15/240) preferred SMS, and the remainder had no preference (Table 3).

A total of 80.4% (193/240) of the participants were willing to use a toll-free health call center or hotline numbers for obtaining health-related information of their interest; the reported reasons for not wanting to use these methods were related to cultural and gender issues, or reluctance/fear to talk to health professionals. Regarding the factor associated with openness to receive health information via a mobile phone, those who owned a mobile phone and those who used it routinely were more likely to be open to receiving information in comparison with those who did not own or use one routinely. However, these associations were not significant after adjusting for other variables. Other factors, including literacy, residency, and age, were also not significantly associated with respondents’ willingness to receive health information via a mobile phone (Table 5).

### Usage of Mobile Phone for Medical Reminders

Approximately 72.1% (173/240) of the participants were willing to receive reminders for National Immunization Days, whereas 89.6 (215/240) wanted to receive reminders for vaccinations and antenatal care visits during pregnancy, as well as reminders about their children’s vaccination schedules.

More than half (120/225, 53.3%) of the participants wanted the reminders 2 days before the scheduled date, whereas 38.2% (86/225) preferred to receive these reminders 1 day before. Only few respondents wanted to receive reminders on the day of immunization (10/225, 4.4%) or 1 week before the scheduled date (9/225, 4.0%). Voice calls were the most preferred method to receive the reminders (188/235, 80.0% of respondents; Table 3).

**Table 4:** Factors associated with perceptions about the use of mobile phones for supporting health (N=240). OR: odds ratio.

Variables		Can mobile phone be used to support health?		Crude OR (95% CI)	Adjusted OR (95% CI)
		No	Yes		
**Residence, n (%)**					
	Rural	39 (32.5)	81 (67.5)	1	1
	Urban	28 (23.3)	92 (76.7)	1.58 (0.90-2.80)	1.22 (0.59-2.52)
**Ability to read, n (%)**					
	No	66 (36.1)	117 (63.9)	1	1
	Yes	1 (1.8)	56 (98.2)	31.59 (4.27-233.48)	20.11 (2.61-155.15)
**Ownership of mobile phone, n (%)**					
	No	41 (41.8)	57 (58.2)	1	1
	Yes	26 (18.3)	116 (81.7)	3.21 (1.79-5.76)	2.23 (1.07-4.61)
**Routine use of mobile phone, n (%)**					
	No	12 (60.0)	8 (40.0)	1	1
	Yes	26 (18.3)	116 (81.7)	4.50 (1.75-11.58)	2.61 (0.87-7.86)
**Age group (years),** **n (%)**					
	32 or older	43 (39.8)	65 (60.2)	1	1
	31 or younger	21 (16.8)	104 (83.2)	3.28 (1.79-6.01)	3.32 (1.56-7.06)
Duration of use (years), mean (SD)		3.73 (1.758)	4.55 (1.971)	1.27 (1.07-1.51)	1.32 (1.08-1.61)

**Table 5:** Factors associated with willingness to receive health information via a mobile phone (N=240). OR: odds ratio.

Variables		Willing to receive health information via a mobile phone		Crude OR (95% CI)	Adjusted OR (95% CI)
		No	Yes		
**Residence, n (%)**					
	Rural	20 (16.7)	100 (83.3)	1	1
	Urban	11 (9.2)	109 (90.8)	1.98 (0.91-4.34)	1.96 (0.77-5.04)
**Ability to read, n (%)**					
	No	28 (15.3)	155 (84.7)	1	1
	Yes	3 (5.3)	54 (94.7)	3.25 (0.95-11.13)	2.25 (0.61-8.37)
**Ownership of mobile phone, n (%)**					
	No	21 (21.4)	77 (78.6)	1	1
	Yes	10 (7.0)	132 (93.0)	3.60 (1.61-8.04)	2.37 (0.95-5.93)
**Routine use of a mobile phone, n (%)**					
	No	8 (40.0)	12 (60.0)	1	1
	Yes	23 (10.5)	197 (89.5)	5.71 (2.12-15.42)	2.82 (0.94-8.47)
**Age group (years), n (%)**					
	32 or older	16 (14.8)	92 (85.2)	1	1
	31 or younger	15 (12.0)	110 (88.0)	1.28 (0.60-2.72)	1.02 (0.39-2.65)
Duration of use (years), mean (SD)		4.78 (2.430)	4.29 (1.886)	0.88 (0.71-1.10)	0.88 (0.71-1.09)

## Discussion

The recent growth in mobile phone apps and the number of mobile phone subscribers in Afghanistan provides an opportunity for applying mobile technology to support health care in the country. Currently, mHealth initiatives have been adopted and reported to be successful in improving health worldwide [2]. Given a high penetration rate, mobile phones can be an effective medium for transmitting health information to the population, particularly among vulnerable groups, particularly women, who may have fewer opportunities to access health information in general. However, the success of mHealth initiatives may vary across different populations. Low literacy and technical capacities are considered a barrier for mHealth adoption [2]. It is therefore critical to understand the baseline perceptions and needs of particular users before initiating an mHealth program. This study explored the potential of using mobile technology to support maternal and child health among Afghan women.

The literacy rate among the women in our study was 24%, which is higher than the national literacy rate for adult females (17%) in Afghanistan [4]. However, the national literacy rate includes older women, many of whom are illiterate; the target population in our study comprised only women of a child-bearing age. Although only half of the women in our sample owned a mobile phone, more than 90% of them had access to a mobile phone and had routinely used one for an average of 4 years. This high accessibility means there is great potential in using mobile phones as a medium for health education.

Only 20 out of 240 women in this study reported that they had never used a mobile phone. A previous study described cultural restrictions and lack of permission for women regarding the use of mobile phones in Afghanistan [5]; however, this reason was only reported by 9 respondents in this study. The main reason for not using a mobile phone was poverty, which was reported by 12 women living in rural areas and 4 in urban areas.

The characteristics of mobile usage were not significantly different between women living in rural areas and those in urban areas, except for ownership of a mobile phone. This may be attributable to the common cultural and social values shared by women in both the urban and rural areas in the province. However, the difference in mobile phone ownership reflects the socioeconomic difference between rural and urban areas.

The majority of our respondents were not familiar with IVR. The effectiveness of using IVR for health education remains uncertain according to the literature. Some studies show the effectiveness of IVR [14], whereas others describe users’ technical difficulty in using it [15]. A study in Uganda showed that training a target population on how to use IVR before project implementation was possible only for small projects [15]. For larger programs, the alternative of using SMS/voice calls may be more effective.

The radio is still the most used and reliable source of news and information in Afghanistan [16], and most mobile phones can support FM radio; more than half of our respondents use mobile phones to listen to the radio. Women’s perceptions of mHealth were assessed. About three quarters of the respondents responded positively. This perception was not significantly different between the urban and rural population, but was significantly different for other demographic characteristics. The literacy rate and ownership of a mobile phone were strongly associated with positive perceptions. This suggested that improving the literacy rate may have an impact on the success of mHealth implementation. Younger women were also more likely to perceive usefulness of mHealth compared with older women, consistent with a national survey conducted in Kenya [17]. This is likely because younger generations are generally more familiar with mobile technology compared with older people.

Majority of the participants were open to receiving health-related information on their mobile phone. This openness was associated with literacy, mobile phone ownership, and routine use of mobile phones. Literate women were 3.6 times more likely to be open to receiving health-related information on mobile phones in comparison with illiterate women. This suggests that mHealth initiatives in Afghanistan could increase, as literacy rates are improving in the country, especially among women. Very few of our respondents were not open to receiving such information, and the reported reasons were mostly related to culture and gender.

Although SMS is the globally preferred method for receiving health-related messages [2], our study showed different results. Most of our respondents preferred voice calls for receiving health-related information and medical reminders. It is obvious that individuals with a low literacy level prefer voice calls in comparison with SMS and/or IVR. For the effectiveness of any mHealth initiative, it is important to consider the end users and target population’s literacy level and preferred type of communication for receiving health-related information and medical reminders.

SMS is the globally preferred method and it is considered to be nonintrusive, as the receivers can read the messages at their convenience instead of answering a call [2,9]. A study conducted in Karachi, Pakistan, shows that SMS reminders were useful for tuberculosis patients in terms of treatment adherence; however, that study also suggested that a two-way reminder system might better support patients in medication adherence in low-literacy populations [18]. A study from rural areas of India also reported that voice calls were preferred in comparison with text messages for medical reminders and health promotion messages [9]. A study conducted in Kenya also observed that telephone call was a more preferable mode of communication among women living in remote areas, whereas women in nonremote areas were likely to prefer text message as their mode of communication [19]. Considering the low literacy rate among Afghan women, voice calls should be the most effective form of communication for an mHealth initiative. However, this may change if literacy rates continue to improve.

The frequency of health-related message delivery is an important issue to be considered while adopting mHealth initiatives. More frequent messages may result in intervention fatigue [13]. Our study showed that more than half of our respondents preferred to receive messages on a weekly basis, rather than daily or monthly.

Medical reminders can play a significant role in treatment/vaccination adherence. A study in rural Kenya showed that mobile phone–based strategies are useful for delivering reminders to target groups. Text message reminders can effectively increase immunization coverage [20]. In addition, a systematic review study documented that text messaging increased adherence to antiretroviral therapy and smoking cessation [21]. In this study, approximately 90% of the women were willing to receive vaccination reminders. Unlike previous studies, most of our respondents preferred to receive voice call reminders rather than text messages. More than half of our respondents preferred to receive reminders 2 days before the appointment date. Because some respondents live far from health facilities, they would need to plan their visit after receiving the reminder. According to national statistics, only 47% of Afghan children under 1 year of age receive the third dose of the Penta III vaccine (hepatitis B and influenza type B vaccines are added to Diphtheria-Pertussis-Tetanus (DPT) vaccine, and are administered in a single-dose vial) [7]. The World Health Organization also reported that the DPT1 to DPT3 vaccination dropout rate in Afghanistan was higher than 15% [22]. Therefore, an mHealth initiative for vaccination reminders, customized for this specific target group, would be one of the effective methods to increase vaccination coverage in the country.

This study was conducted in one province of Afghanistan, which may not represent all women living in the country, especially those in very remote regions or conflict areas. In addition, our study areas contained at least one mobile network, and most of women in this study had used mobile phones for an average of 4 years. There are still some areas in Afghanistan with no available networks that have only recently obtained a network; perceptions of using mobile phones for supporting maternal and child health may differ among women who are new to mobile phone use. Further studies are needed to assess mHealth perceptions among this group of women.

Our study explored the perceptions of women regarding the use of mobile phones for maternal and child health in Afghanistan. This preintervention assessment yielded valuable information for the design and implementation of mHealth interventions. Although the findings in this study are encouraging in terms of the potential benefits of adopting mHealth interventions in Afghanistan, all the end users’ concerns should be considered and intervention designs should be contextualized according to the target population.

## Abbreviations

DPT: Diphtheria-Pertussis-Tetanus
IVR: interactive voice response
OR: odds ratio
SMS: short message service
